# Digital and Immersive Technologies for Rehabilitation in Complex Psychosis: State of the Art and Future Directions

**DOI:** 10.3390/medicina62040765

**Published:** 2026-04-15

**Authors:** Giuseppe Marano, Mariateresa Acanfora, Giuseppe Mandracchia, Gianandrea Traversi, Osvaldo Mazza, Antonio Pallotti, Giorgio Veneziani, Carlo Lai, Emanuele Caroppo, Marianna Mazza

**Affiliations:** 1 Department of Neuroscience, Head-Neck and Chest, Section of Psychiatry, Fondazione Policlinico Universitario Agostino Gemelli IRCCS, Largo Agostino Gemelli 8, 00168 Rome, Italy; mariatacanfora@gmail.com (M.A.);; 2 Department of Neuroscience, Section of Psychiatry, Università Cattolica del Sacro Cuore, 00168 Rome, Italy; 3 Unit of Medical Genetics, Department of Laboratory Medicine, Ospedale Isola Tiberina-Gemelli Isola, 00186 Rome, Italy; gianandrea.traversi@gmail.com; 4 Spine Surgery Department, Bambino Gesù Children’s Hospital IRCCS, 00168 Rome, Italy; osvaldo.mazza1973@hotmail.it; 5 Department of Management and Law, University of Rome “Tor Vergata”, 00133 Rome, Italy; 6 Department of Dynamic and Clinical Psychology, and Health Studies, Sapienza University of Rome, 00185 Rome, Italy; 7 Department of Mental Health, Local Health Authority ASL Roma 2, 00159 Rome, Italy; emanuele.caroppo@aslroma2.it

**Keywords:** psychosis, complex psychosis, rehabilitation, digital technologies

## Abstract

Complex psychosis (CP) remains one of the most challenging conditions in mental health, characterized by persistent symptoms, cognitive impairment, functional disability, and reduced autonomy. Traditional rehabilitation approaches, although essential, are often insufficient to address the multidimensional needs of these individuals. Over the past decade, rapid advances in digital health have opened new opportunities to enhance psychosocial rehabilitation, improve engagement, and personalize treatment pathways. This narrative review synthesizes current evidence on the use of digital and immersive technologies in the rehabilitation of people with CP, including virtual reality (VR), augmented reality (AR), telerehabilitation platforms, mobile health (m-Health) applications, digital phenotyping, and AI-assisted cognitive remediation. We examine clinical trials, feasibility studies, and real-world implementations published between 2015 and 2025, highlighting the efficacy of VR-based social cognition training, remote cognitive remediation, ecological momentary interventions, and hybrid digital–in-person rehabilitation models. Mechanisms of action, transfer to real-world functioning, and predictors of engagement are described. Barriers such as digital literacy, access disparities, privacy concerns, and clinical integration are critically discussed. We also outline future directions, including adaptive algorithms, biosensor integration, and the development of multimodal digital ecosystems tailored to individual recovery trajectories. By integrating technological innovation with recovery-oriented care, digital rehabilitation tools have the potential to transform the treatment landscape for people with CP. This review offers a roadmap for clinicians, researchers, and policymakers seeking to incorporate evidence-based digital solutions into modern psychiatric rehabilitation.

## 1. Introduction

Psychosis can include schizophrenia, mood disorders with psychotic features, delusional disorder, active delirium, and neurodegenerative disorders accompanied by various psychotic symptoms [1]. The term “complex psychosis” (CP) refers to the primary diagnosis of a psychotic illness, which includes schizophrenia, bipolar affective disorder, psychotic depression, delusional disorders, and schizoaffective disorder, with severe, treatment-resistant psychotic symptoms and functional impairment. It is important to note that “complex psychosis” is not a universally recognized diagnostic category but rather a service-oriented construct used to describe individuals with severe, treatment-resistant psychotic disorders and high care needs. This term is commonly adopted in clinical service frameworks to guide rehabilitation planning rather than to define a specific nosological entity. In the present review, the term complex psychosis is used pragmatically and from a rehabilitation-oriented perspective, rather than as a diagnostically homogeneous or pathophysiologically unified entity. The rationale for grouping schizophrenia-spectrum disorders, bipolar disorder with psychotic features, psychotic depression, and related conditions lies in the shared presence of severe functional impairment, persistent symptoms, cognitive difficulties, reduced autonomy, and the need for intensive, multimodal rehabilitation pathways.

People with CP have one or more of the following symptoms: cognitive deficits; coexisting mental health conditions, including substance abuse; preexisting neurodevelopmental disorders, such as autism spectrum disorder (ASD) or attention deficit hyperactivity disorder (ADHD); physical health problems, such as diabetes or cardiovascular disease [2]. In general, therefore, health treatments are based on pharmacological guidelines, psychological and family interventions, and staff training. Physical healthcare is part of rehabilitation and is based on a family doctor, a professional who ensures continuity of physical healthcare between different contexts, on the promotion of a healthy lifestyle on monitoring of physical conditions with routine examinations and on early identification of health risks. All these problems affect the person’s daily functioning and require a period of rehabilitation to enable recovery and ensure that the optimal level of independence is achieved.

People with CP are those who are currently hospitalized outside the area for rehabilitation; have recurrent or prolonged hospitalizations, e.g., longer than 60 days, in acute psychiatric wards; live in facilities with 24 h staffing; are currently under the care of forensic mental health services; receive assistance from early intervention services for psychosis; are physically frail and may need placement with specialist support; are young adults transitioning from child and adolescent mental health services to adult mental health services [3].

**Figure 1:**
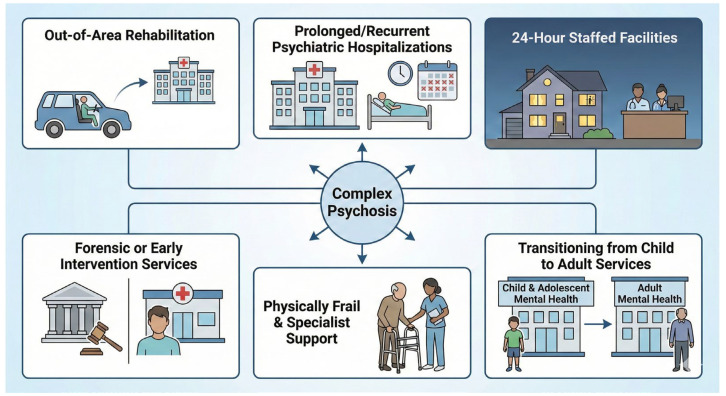
Clinical and care pathways defining complex psychosis. Note: Individuals with CP include those undergoing out-of-area rehabilitation, experiencing prolonged or recurrent psychiatric hospitalizations, living in 24 h staffed facilities, receiving care from forensic or early intervention psychosis services, physically frail individuals requiring specialist support, and young adults transitioning from child and adolescent to adult mental health services.

Concluding, people with CP can be considered a clinically heterogeneous group characterized by severe functional impairment and high care needs, including those undergoing out-of-area rehabilitation, prolonged hospitalizations, or supported living arrangements, as summarized in Figure 1.

Rehabilitation interventions are based on promoting the development of daily living skills, providing practical activities to improve personal hygiene, housekeeping, use of money, use of transportation and communication, including digital communication. These interventions have many purposes: creating a personalized plan, which is reviewed frequently as needed; offering structured group activities to improve interpersonal skills, with constant review and individual support from an assigned staff member; involvement in community activities, such as study, work, and leisure, to structure and give purpose to the person’s day, to obtain qualifications, and for those who want to work, offering supported employment, transitional jobs, or volunteering; assessing the person’s use of illicit substances and working with specialist substance abuse services where necessary [4]. The limitations of rehabilitation are essentially linked to practical implementation, the complexity of the disorder itself, and resource management.

CP is linked to negative and cognitive symptoms, which reduce the person’s ability to actively participate in programs. CP often involves a lack of response to medication, with the subsequent need to increase doses or use multiple medications together, resulting in increased side effects and the need for close clinical monitoring [5]. Learning skills in a protected environment can be difficult to transfer to everyday life. This is complicated by the presence of comorbidities, such as substance abuse or other psychiatric disorders. The availability of resources affects the effectiveness of rehabilitation: the lack of local facilities has a long-term impact; the multidisciplinary approach required needs highly competent staff who are continuously trained; the coordination of the various figures and facilities requires robust communication and recording systems [6].

Digital technologies may offer innovative tools to overcome the limitations of traditional rehabilitation, improving access, effectiveness, and adherence to treatments [7]. This narrative review summarizes the current evidence on the use of digital technologies in rehabilitation. Given the rapid expansion of digital technologies in psychiatry, this review adopts a broad, integrative perspective aimed at mapping current approaches and identifying shared mechanisms, rather than providing an exhaustive analysis of individual technologies.

## 2. Conceptual Framework: Digital Rehabilitation in Severe Mental Illness

### 2.1. Recovery-Oriented and Patient-Centered Models

Rehabilitation is based on the concept of recovery, which consists of leading a productive and satisfying life even in the presence of the limitations imposed by mental illness. According to the Substance Abuse and Mental Health Services Administration (SAMHSA), recovery is supported by four dimensions: health, the ability to manage one’s illness; home, the presence of a safe place to live; purpose, the existence of meaningful activities, such as work, study, leisure; community, feeling part of a social network [8]. Rehabilitation is a set of individualized projects aimed at enhancing functioning in the individual’s living environment, with as little professional support as possible, maximizing functional independence and preventing the onset of secondary complications.

Rehabilitation interventions must have a multidimensional perspective, i.e., consider the biological, psychological, and social components of the person [9]. Rehabilitation services for people with CP should be integrated into a comprehensive local mental health care service. These services should be offered in as non-restrictive a setting as possible and should aim to progress people from more intensive support to greater independence through rehabilitation, recognizing that not everyone will return to the same level of independence they had before their illness and may need long-term supported housing [10]. Non-pharmacological treatments include cognitive remediation (CR) interventions, either paper-based or computerized.

Psychiatric rehabilitation activities and techniques are divided into healthcare rehabilitation activities, which include diagnostic, therapeutic, and assessment interventions aimed at minimizing limitations in abilities and symptoms, and social rehabilitation activities, which include interventions and actions to ensure maximum participation in the individual’s social life [11].

### 2.2. Digital Intervention Categories

The generation born after 1990 is defined as “digital natives,” able to find information and digital stimuli quickly and easily from digital platforms [12]. Evidence suggests that individuals with psychosis also use electronic devices, digital applications, and social media resources in a manner comparable to those without this condition and are interested in using them for their health [13].

Patients consider technology less stigmatizing, more flexible, and capable of promoting empowerment. The study by Camacho and Torous also reveals that most staff and clinicians expressed a desire and interest in receiving training in digital skills and believe that technologies can be easily integrated into their work: education and family support are considered the areas most amenable to improvement with technologies [14]. The key technological areas to highlight are: information and psychoeducation, such as websites and apps that offer resources to patients and their families; monitoring systems, which enable patients to identify symptoms and doctors to obtain objective information; direct interventions, such as the use of peer social platforms or clinical interventions, specialized apps aimed at specific objectives such as emotional regulation, cognitive behavioral therapy (CBT), or interviews; and advanced technologies, such as augmented reality (AR) or virtual reality (VR) [15].

Rapid advances in digital health may create new opportunities to enhance rehabilitation for patients with CP, increase patient engagement, and personalize therapeutic pathways (Table 1).

### 2.3. Mechanisms of Action and Neurocognitive Targets

Cognitive impairment is an important feature of the disease, which begins even in the premorbid phase. The presence of cognitive impairment is a negative predictor of the individual’s social and occupational functioning: it is therefore essential to treat it as early as possible. This is why CR techniques exist, based on the recovery model, which aim to improve and restore the most affected cognitive domains, such as attention, memory, executive functions, working memory, reasoning, problem solving, and processing speed, using strategies learned during the interventions carried out [16].

The largest meta-analysis on the subject conducted by Vita et al. highlighted that CR produces significant and lasting improvements in overall cognition and in everyday life, also acting on negative symptoms [17].

CR techniques are interventions based on behavioral training. These techniques are divided into: reparative, i.e., based on neuroplasticity, i.e., brain adaptation to internal or external stimuli and repeated and continuous exercise, promoting damage repair; compensatory, i.e., circumventing the deficit, compensating for it with strategies that use the subject’s residual cognitive abilities and the resources of the surrounding environment; modulatory, i.e., modifying the processes that lead to problematic and stressful behaviors [18]. Having an experienced therapist who conducts CR may contribute to enhance cognitive and functional gains [19]. CR incorporated into the patients’ rehabilitation programs allow them to use and consolidate the cognitive gains learned in everyday life [20]. For example, CBT identifies recurring dysfunctional thoughts and patterns, with the aim of replacing or integrating them with more functional patterns [21].

The approaches used in CR techniques are bottom-up, i.e., from the recovery of basic skills to more complex skills, and top-down, i.e., the acquisition of more complex skills used to improve basic domains and functions [22]. The areas that benefit most from CR techniques are: visuospatial memory, in patients with a higher number of hospitalizations and more severe pathology; working memory, in patients with lower levels of apathy, social withdrawal, and affective flattening; verbal memory and cognitive flexibility, in patients with more severe cognitive deficits, probably due to the higher margin for improvement [23]. Therefore, age and how much time has passed since the onset of the disease are not considered as limitations in the use of CR [10].

**Figure 2:**
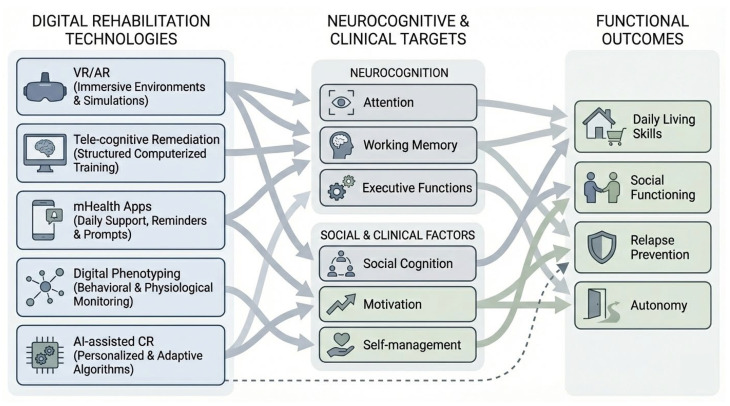
Mechanisms of action and functional targets of digital rehabilitation technologies in complex psychosis. Note: Digital interventions act on specific neurocognitive and clinical domains, including attention, working memory, executive functions, social cognition, motivation, and self-management, through distinct technological modalities such as virtual and augmented reality, tele-cognitive remediation, m-Health applications, digital phenotyping, and AI-assisted cognitive training. Improvements in these domains support functional outcomes, including daily living skills, social functioning, autonomy, and relapse prevention, thereby facilitating the transfer of rehabilitation gains to real-world contexts.

The mechanisms through which digital rehabilitation technologies act on neurocognitive domains and functional outcomes are summarized in Figure 2. This figure provides a conceptual framework and is not intended to represent quantitative effect sizes or comparative efficacy.

### 2.4. Literature Search Strategy

This narrative review was conducted through a structured, though non-systematic, search of the literature. The main databases consulted included PubMed, Scopus, and Web of Science. The search strategy combined terms related to psychosis (e.g., “psychosis”, “schizophrenia”, “bipolar disorder”), rehabilitation (e.g., “cognitive remediation”, “psychosocial rehabilitation”), and digital technologies (e.g., “virtual reality”, “augmented reality”, “m-Health”, “digital phenotyping”, “artificial intelligence”, “telepsychiatry”).

Studies published between 2015 and 2025 were considered. Priority was given to randomized controlled trials, systematic reviews, meta-analyses, and well-designed observational studies. Feasibility and pilot studies were included when relevant to emerging technologies. Particular attention was paid to differentiating between levels of evidence, in order to distinguish preliminary findings from more robust evidence. Non-English articles, case reports, and studies not directly related to psychiatric rehabilitation were excluded.

**Figure 3:**
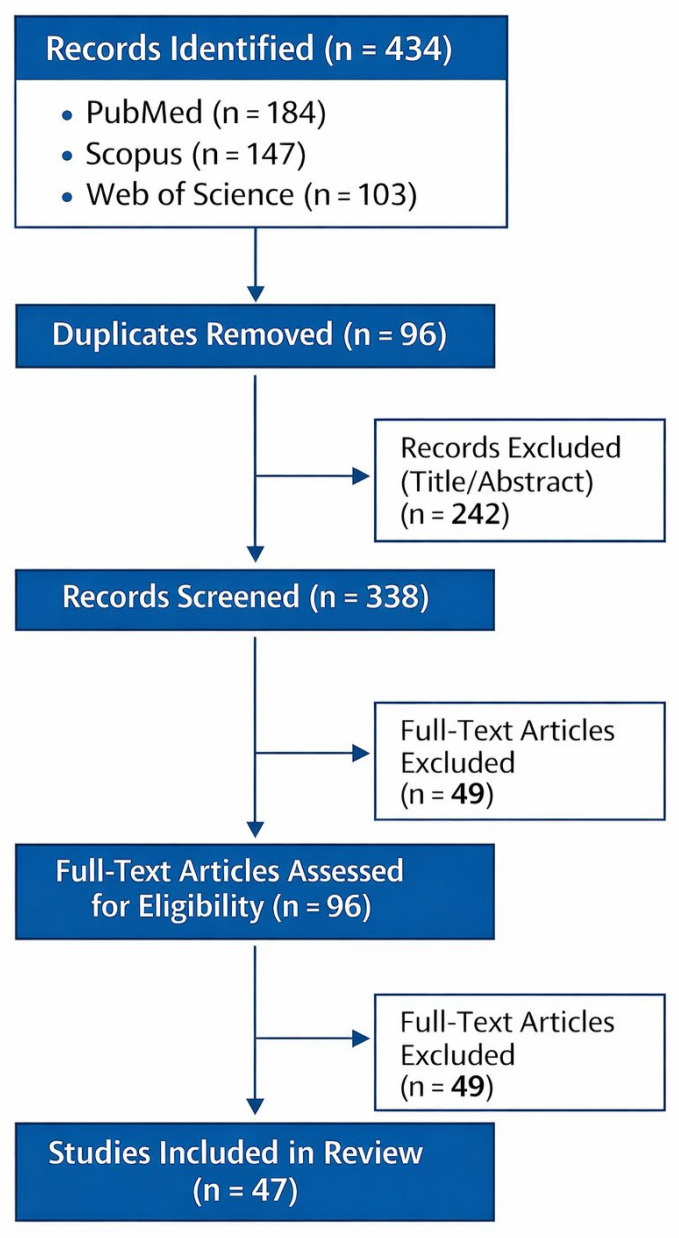
Flow diagram of study identification and selection for this structured narrative review.

The selection of studies was guided by relevance to the topic and conceptual contribution rather than by formal systematic review procedures. Records identified through database searching were screened first by title and abstract for relevance to the topic. Full texts were then assessed for conceptual fit with the aims of the review, with priority given to systematic reviews, meta-analyses, randomized controlled trials, and well-designed observational studies. Pilot and feasibility studies were retained primarily in areas of emerging technology where higher-level evidence remains limited. Because this review was narrative rather than systematic, no formal risk-of-bias tool was applied, and study selection retained an element of interpretative judgment. This approach may have introduced selection bias and limits reproducibility. To improve transparency, the main stages of literature identification, screening, and study selection are summarized in Figure 3.

## 3. Virtual Reality (VR) and Augmented Reality (AR) Interventions

### 3.1. VR for Social Cognition/Functioning and Symptom Management

The negative symptoms of schizophrenia, such as anhedonia, emotional flattening, and social withdrawal, often lead to rumination and social isolation. Negative symptoms are those that respond least to medication. VR is an immersive experience that excludes the physical world [24]. In the field of mental health, Virtual Reality Therapy (VRT) has been used as an integrated intervention within the user’s personalized therapeutic rehabilitation project [25,26]. In particular, two objectives of VRT in healthcare have been identified: VR as a simulation tool and VR as an interaction tool [27]. In the virtual world, users are active participants and not external observers: this interactivity is fundamental to understanding social environments [28]. VR interventions have simulated real environments to facilitate training in social skills (for example in virtual conversations with avatars), cognitive functioning (for example decision-making), functional rehabilitation (for example performing daily tasks) and work recovery (for example job interview simulations) [29,30,31]. VRT is also used as a tool for psychoeducational intervention aimed at recognizing events that precede a crisis or that cause changes in positive symptoms, such as auditory hallucinations, and is also more cost-effective [32,33].

It has been observed that VRT integrated with CBT reduced paranoid and anxious symptoms in social relationships, compared to the two techniques used individually, with benefits maintained at six months [34]. Rus-Calafell and Schneider evaluated improvements in cognitive functioning, particularly in areas such as memory, attention, and planning; social cognition, e.g., emotion recognition and cognitive biases; and work outcomes [35,36].

Although several studies report improvements in social cognition and symptom reduction with VR-based interventions, most trials involve relatively small sample sizes and heterogeneous designs. When compared to traditional interventions, such as cognitive behavioral therapy or social skills training, VR appears to provide comparable benefits, particularly in engagement and ecological validity, but evidence for superiority remains limited and requires larger randomized controlled trials. Reported benefits are more consistent for engagement, social cognition training, and symptom-focused experiential work than for robust long-term functional outcomes. In particular, evidence for transfer to everyday functioning, relapse prevention, or sustained occupational and social recovery remains limited.

### 3.2. AR for Skill Training and Environmental Support

AR superimposes virtual elements onto the real environment in real time [37]. The presence of AR reflects an expansion towards immersive technologies, blending real and virtual elements. In this way, patients are able to explore their reality with the addition of visual, auditory, and sensory stimuli [38]. Current studies on this topic are few and the results are not standardized. Despite this, schizophrenia is one of the fastest-growing fields in psychiatry for the application of AR, as it can act on negative and cognitive symptoms, where medication often fails. AR may bridge this gap by working on social skills, creating controlled environments for practicing social interactions, on symptom management, using avatars or voices to simulate the patient’s perceptions and teach them strategies under therapeutic guidance and on engagement, as technologies similar to “gaming” are particularly attractive to young adults [39]. In this way, AR synchronizes intention and virtual movement to help the individual take more control of their actions, corrects space-time perception to reduce fragmentation and define the boundaries of the self, offers a protected and realistic environment in which to train [40]. AR appears to offer promising benefits, but the evidence base is even more preliminary than for VR, with fewer studies and lower standardization across protocols and outcomes.

## 4. Telerehabilitation Platforms and Hybrid Models (In-Person + Online)

### 4.1. Remote Group Psychosocial Interventions

Group psychosocial interventions are based on self-management of symptoms, working on specific deficits, promoting well-being and recovery, and support groups. Symptom self-management is based on CBT for psychosis, programs targeting social anxiety or emotional regulation for those who have experienced trauma, mindfulness-based programs for managing and accepting auditory hallucinations, programs for positive emotions in schizophrenia, which aim to reduce negative symptoms and increase positive feelings [15]. The specific deficits worked on in groups are usually those of metacognition, social cognition, to work on cognitive biases and social skills, social skills training (SST) to improve socialization and reduce apathy [41].

The effectiveness of SST on negative symptoms is comparable to the effectiveness of CBT on positive symptoms, so SST is specifically effective for what it was developed for, i.e., negative symptoms and social skills [41]. Groups such as “I am super” or “coping and skill” improve self-esteem and problem management, implementing relationships in social and work settings [42]. Support groups, including online groups, may help through sharing experiences, mutual inspiration, and sharing strategies [43]. The advantages of the group are peer learning, socialization, universality through sharing, and altruism among group members.

Improvements in technology and access to high-speed Internet have increased the acceptability of telemedicine services for people with mental disorders, including those on the schizophrenic spectrum [44]. Before the COVID-19 pandemic, telemedicine was used in individual sessions with patients already being treated by the operator when it was impossible for either party to meet in person. Lynch et al. demonstrated that recovery services can be provided virtually to people with CP during the pandemic: in a New York clinic, 90% of patients accepted telemedicine sessions, including group therapy, and were able to follow treatment plans in virtual format [45]. The main advantages are greater accessibility to care, real-time information, reduced stigma, and a greater sense of control on the part of the patient [46]. This particularly improves accessibility for patients for whom social interaction is a symptomatic problem [47].

This has led to the emergence of tele-psychiatry, a specialization of telemedicine that uses telephone calls and video platforms to carry out follow-ups, psychiatric assessments, and rehabilitation programs [48]. Training clinicians on the relationship between social media and mental health facilitates bonding with patients and allows questions about online behavior to be integrated into clinical assessments and rehabilitation treatment plans [49].

### 4.2. Tele-Cognitive Remediation (Tele-CR)

Telemedicine could be useful for permanently integrating CR into healthcare services, as the following factors must be considered: planning, i.e., moving from passive dissemination to a structured plan lasting at least two years; access, i.e., ensuring geographical proximity, including through technology, to prevent dropouts; personnel, i.e., ensuring supervision by expert centers of local centers, including via telematics [50]. Tele-CR has similar costs to face-to-face CR and has a lower social cost, for example, less patient travel in terms of cost and time [51]. The dropout rate is lower than for face-to-face therapy, thanks to the remote nature of the treatment, which is even more appreciated by those who study or work [52].

Cella and Wykes point out that patients have achieved concrete goals in their daily lives, such as reading, socializing, or cooking, thanks to metacognition exercises and strategies to be implemented, consistent with the CR approach, despite there being no striking improvements in classic neuropsychological tests [52]. The success of Tele-CR depends heavily on the therapist, as they normalize the difficulties that patients commonly face due to their condition, resolve connection or software problems, and provide a sense of security in tackling digital exercises [53]. The results of the literature agree that cognitive rehabilitation using a digital platform and the involvement of human support have the possibility to significantly improve outcomes, particularly functional ones.

It is possible to conclude that the current literature on telerehabilitation and Tele-CR suggests good feasibility, acceptability, and continuity of care, particularly in contexts where in-person access is difficult. However, the evidence base is still mixed in terms of study design, sample size, and follow-up duration. Several studies primarily support usability, adherence, and service accessibility, whereas evidence for superiority over face-to-face rehabilitation or for durable functional gains remains less consistent. Moreover, therapist support appears to be a key moderating factor, indicating that the effectiveness of remote interventions may depend less on the digital platform alone than on the quality of human guidance embedded within it.

## 5. Mobile Health (m-Health) Applications

### 5.1. Psychoeducation and Self-Management Apps

Mental Health applications for CP combine structured psychoeducation modules, real-time subjective monitoring tools, medication reminders, and decision support features, with the aim of improving insight, self-management skills, and active participation in rehabilitation programs [54].

Apps designed to promote shared decision-making in the treatment of psychotic disorders have been found to be generally usable and well accepted by patients and clinicians, showing the potential to strengthen the therapeutic alliance and involvement in defining the treatment plan, although data on medium-term clinical outcomes are still preliminary [55].

Blended interventions, which integrate the use of the app into sessions through joint discussion of the data collected (e.g., mood patterns, sleep, activity, symptoms), seem particularly promising for supporting shared decision-making processes and making the link between treatment goals and the person’s daily life more transparent. Platforms that offer intuitive visual feedback and customizable pathways (e.g., choice of modules on sleep, activity, recovery, substance use) facilitate the adaptation of the intervention to patient preferences and can increase the sense of agency with respect to one’s own treatment pathway.

At the same time, the real effectiveness of these apps depends on adequate support structures (training of clinicians, guidance in use, management of possible negative reactions), which are crucial for their stable integration into routine clinical practice [55].

### 5.2. Symptom Monitoring and Relapse Prevention

The combined use of Ecological Momentary Assessments (EMA) and passive sensors (GPS for mobility, microphone for conversations) via smartphones allows for fine-grained monitoring of social activity and loneliness in people with schizophrenia, reducing recall bias and providing objective indicators of social functioning [56].

In support of this, the EMPOWER trial (randomized study *n* = 73 patients with high-risk schizophrenia) demonstrated the feasibility of an integrated intervention using and combining apps for early warning signs, peer support, and clinical assessment. The results showed that approximately 71% maintained adequate adherence to monitoring for 12 months (mean 31.5 weeks), as well as a reduction in fear of relapse compared to standard treatment (mean effect d = 0.53) and an acceptable safety profile (29 adverse events, only 13 related to the app) [57].

Consumer wearables (Fitbit, Samsung) confirm correlations between steps, heart rate variability, and sleep with symptom severity, identifying potential markers for preventing relapses but highlighting the need for standardization [58].

### 5.3. Apps for Motivation and Daily Functioning

A second family of m-Health interventions uses video games on smartphones to influence motivation, behavioral activation, and cognitive functions, with positive effects on social functioning and daily activities.

Recent randomized studies on smartphone video games in patients with chronic schizophrenia report significant improvements in memory and attention, a reduction in negative symptoms, good tolerability, a low risk of “gaming addiction,” and the possibility of integration into rehabilitation programs [59].

Furthermore, digital phenotyping via smartphone (mobility, environmental voice interactions) is associated with indicators of loneliness and social functioning, suggesting that these apps may support ecological goals such as time spent outside the home, frequency of contact, and meaningful activities [56].

### 5.4. Evidence for Usability and Effectiveness

Recent literature shows that m-Health apps for psychosis achieve adherence rates > 80% in the first few weeks, with 71% of users maintaining “clinically useful” use (>33% daily monitoring) for months, thanks to reminders, peer support, and clinical triage. However, effects on “hard” outcomes (relapses, hospitalizations, overall functioning) are heterogeneous, limited by small samples and short follow-ups; the most robust evidence is associated with process outcomes (self-management, insight, adherence, cognitive insight, reduction in fear of relapse) [57].

The available data confirms that the use of m-Health should be considered a promising complement, but not a substitute, for multidimensional rehabilitation in psychosis, with good usability/acceptability [58].

Taken together, m-Health interventions in psychosis show encouraging signals in relation to self-management, monitoring adherence, usability, and patient engagement. Nevertheless, the literature is characterized by small samples, heterogeneous intervention formats, variable endpoints, and frequently short follow-up periods. As a result, the strongest evidence currently concerns process outcomes, such as symptom monitoring, insight, treatment participation, and perceived support, whereas evidence for harder outcomes, including relapse reduction, hospitalization prevention, or sustained improvement in real-world functioning, remains more limited and inconsistent.

## 6. Digital Phenotyping and Sensor-Based Monitoring

It is important to clarify that digital phenotyping, although highly promising, should not currently be considered a validated early-warning system for relapse in psychosis. Most existing models rely on associative patterns derived from behavioral and physiological data, and their predictive performance has been demonstrated primarily in exploratory or pilot studies. While these approaches can identify deviations from individual baselines and generate clinically relevant signals, the translation of such signals into reliable, actionable predictions remains limited. Therefore, digital phenotyping should be interpreted as an emerging monitoring framework rather than a fully established predictive tool, requiring further validation through large-scale, prospective, and real-world studies before routine clinical implementation.

### 6.1. Passive Sensing

Passive detection forms the methodological basis of digital phenotyping in psychotic disorders, enabling data collection based on three behavioral dimensions: movement, sleep, and social interaction, without any explicit burden on the patient [60].

Accelerometric measurement by smartphones provides objective quantification of motor activity. In fact, accelerometric markers have been shown to correlate significantly with gold-standard measures of clinically assessed negative symptoms. Phone inactivity and wearable actigraphy data allow for robust estimation of sleep duration and quality, parameters that are strongly correlated with symptom severity. A multicenter study has documented a substantial correlation between smartphone-derived sleep duration and performance on standardized cognitive tests. Sociality is quantified through the extraction of behavioral features from anonymized communication logs [61,62]. It has been shown that abnormalities in habitual communication patterns were a particularly sensitive predictor of imminent relapses, manifesting themselves with an increased incidence two weeks prior to hospital admission.

The synergistic integration of these three behavioral domains considerably enhances the overall predictive signal: when abnormalities appear simultaneously in movement patterns, sleep architecture, and social characteristics, the diagnostic specificity for imminent psychiatric decompensation may improve predictive accuracy, although current evidence remains preliminary and largely associative. Furthermore, these passive markers have high adherence rates for this vulnerable population [63].

### 6.2. Predicting Relapse Trajectories

Predicting relapse trajectories is the most relevant clinical application of digital phenotyping, as it transforms passive markers into sophisticated computational algorithms. Barnett et al. developed anomaly detection models that establish a personalized baseline of individual behavior during phases of clinical stability, subsequently identifying statistically significant deviations from that baseline, reporting behavioral anomalies that precede overt relapse by 9–14 days. This critical time window offers a realistic opportunity for layered preventive interventions: intensification of clinical monitoring, enhancement of psychosocial therapy, or consideration of intensification of drug therapy prior to the acute crisis [63].

A recent study has shown that using encoder–decoder neural network architectures applied to passive smartphone data, a median increase of 108% in the incidence of behavioral abnormalities was documented during the 30-day period prior to clinical relapse. The simultaneous integration of multiple passive markers provides methodological robustness that no single marker could provide on its own.

Unresolved epistemological questions remain regarding causality versus association between detected anomalies and relapse, requiring prospective randomized controlled trials to establish the true clinical efficacy of anomaly detection-based intervention [64]. In the future, a new healthcare management model using digital devices should be integrated with the approach of digital phenotyping and personalized mobile interventions [65].

### 6.3. Ecological Momentary Assessments (EMA)

Ecological Momentary Assessments (EMA) allow for repeated sampling in real time of the patient’s subjective experience in the context of daily life, including psychotic symptoms, affective states, and stress in the psychosocial context. EMA studies in psychotic populations show good feasibility but also highlight a significant daily burden, with intensive protocols requiring careful planning of frequency and duration. In this context, digital EMAs are described as a useful real-time monitoring tool to be integrated into broader care models that include other assessment methods and clinical contact. In conclusion, EMAs should be designed as a complement to passive monitoring and clinical contact [66,67].

Although digital phenotyping represents one of the most innovative areas in contemporary psychiatric research, the current evidence remains largely exploratory. Most available studies are observational, proof-of-concept, or based on associative modeling rather than confirmatory clinical validation. These approaches may help identify behavioral deviations or clinically relevant signals, but their predictive utility for relapse and their readiness for routine clinical implementation remain insufficiently established. At present, digital phenotyping should therefore be regarded primarily as an emerging monitoring framework rather than a validated standalone predictive tool.

## 7. Artificial Intelligence-Assisted (AI-Assisted) and Gamified CR

### 7.1. Adaptive Algorithms and Personalized Training

The computerized cognitive remediation (CCR) programs used employ dedicated software with repeated exercises on memory, attention, and executive functions, showing significant improvements in cognitive and negative domains in chronic patients with schizophrenia [68,69]. Some programs use algorithms that adapt to patient performance; specifically, when accuracy reaches 80%, the system automatically increases the difficulty of the tasks, as demonstrated in CCR programs that improve cognitive function, negative symptoms, and serum glial derived neurotrophic factor (GDNF) levels in chronic schizophrenia patients. This approach surpasses traditional static protocols, reducing the need for constant clinical supervision and adapting to the heterogeneity of deficits, such as memory and cognitive flexibility [68,69,70].

### 7.2. Gamification Strategies for Engagement

Gamification strategies integrate structural playful elements (points, levels, immediate feedback, avatars) to increase engagement and reduce dropouts in CR programs, especially for schizophrenia. To this end, a tool called ThinkTactic Virtual Reality has been developed iteratively with psychotic and clinical patients, allowing them to experiment in immersive environments (navigating a city, apartment, restaurant) simulating real tasks, overcoming abstract exercises, and promoting transfer to everyday life [71].

Using adaptive algorithms, it balances challenge and feasibility, avoiding boredom and frustration: the VR Coach delivers CR strategies with instant feedback. It should be mentioned that the complexity of the interface could generate friction, so it is necessary to prioritize simplicity and a gradual learning curve [71].

Game-based therapy has shown encouraging results for selected cognitive deficits in adults with schizophrenia, often with good adherence, but the available evidence remains preliminary [72,73]. Immersive VR has shown preliminary promise in targeting cognitive functions, although methodological standardization and larger confirmatory studies are still needed [72].

### 7.3. Transfer to Daily Living and Functional Outcomes

The critical issue in CR for psychotic disorders remains the transfer of the skills practiced to everyday life contexts, an area in which approaches based on ecological virtual environments and functional goals show good potential, albeit still partial [69,70,71].

In developing ThinkTactic VR, participants with experience of psychosis emphasized that realistic scenarios involving navigating the city, using public transport, shopping at the supermarket, and interacting in an apartment or restaurant are not simply a “side dish” of CR, but a prerequisite for imagining the transfer of acquired skills into real life [71]. In this program, cognitive tasks (problem solving, memory, emotional regulation, theory of mind) are integrated directly into everyday activities, with the support of a virtual coach and a therapist who concludes the sessions with brief “bridging” discussions aimed at connecting the strategies to the patient’s personal routine and recovery goals [71].

A retrospective analysis of CCR Therapy in patients with chronic schizophrenia indicates that short treatment cycles are associated with robust improvements in both executive control indices and inpatient social functioning scales, with a marked increase in daily living skills, motivation, and social skills compared to drug treatment alone [69]. Despite the growing sophistication of digital rehabilitation tools, the transfer of cognitive and behavioral gains to real-world functioning remains a critical and only partially resolved challenge. While digital modalities, particularly immersive environments and gamified cognitive training, are effective in targeting specific neurocognitive domains under controlled conditions, their impact on everyday functioning, such as social participation, occupational performance, and independent living, is often modest. This gap may reflect the limited ecological validity of some interventions, as well as the complexity of translating structured task performance into dynamic real-life contexts.

Evidence suggests that transfer is more likely to occur when digital interventions are embedded within broader rehabilitation frameworks, including therapist support, goal-oriented training, and explicit “bridging” strategies that connect digital exercises to patients’ daily routines.

Hybrid models combining digital tools with in-person rehabilitation appear particularly promising in enhancing generalization. However, current data remain heterogeneous, and many studies rely on short-term outcomes or surrogate cognitive measures rather than robust functional endpoints. Future research should prioritize longitudinal designs and standardized measures of real-world functioning to better assess the true clinical impact of digital rehabilitation approaches.

### 7.4. Comparative Efficacy vs. Traditional CR

A long-term follow-up (24 months) compared the effects of CCT and manual cognitive remediation (MCT) on 85 patients with schizophrenia, demonstrating that both approaches maintain significant cognitive improvements in attention, verbal memory, and executive functions compared to standard treatment, with no substantial differences between modalities. CCT stands out for its greater efficiency (less supervision time) and adherence, while MCT better promotes functional transfer thanks to personalized therapeutic interaction. Both reduce negative symptoms and improve overall functioning, confirming the lasting effectiveness of CR in real clinical settings [74].

Overall, current evidence suggests that digital cognitive remediation may improve cognitive outcomes, with potential advantages in scalability, accessibility, and patient engagement, although functional transfer may still benefit from therapist-mediated interventions.

AI-assisted and gamified cognitive remediation strategies appear particularly promising in terms of personalization, engagement, and scalability. However, the evidence remains uneven across intervention types, with many reports still based on feasibility studies, pilot trials, or small controlled studies rather than large confirmatory randomized trials. Improvements are most consistently reported in selected cognitive domains and user engagement, while evidence for durable ecological transfer and broader functional recovery remains less robust. These findings suggest that such technologies may best be understood, at present, as promising adjuncts to rehabilitation rather than fully established standalone interventions.

## 8. From Technology to Practice: Implementation, Equity, and Ethics

### 8.1. Digital Inclusion and Disparities

The large-scale implementation of digital interventions for schizophrenia risks widening the health and socioeconomic gaps that already exist among people with the disorder, a population in which poverty is highly endemic and access to technology remains stratified. Supporting this is a French national study of 916 patients with stable schizophrenia, 80.7% of whom lived below the poverty line, with significant inequalities in access to non-pharmacological services and levels of social integration, despite universal health coverage guaranteed by the public system [75].

Epidemiological studies on the digital divide indicate that patients with schizophrenia characterized by low educational attainment, occupational instability, and limited economic resources are significantly less likely to use social media, smartphone applications, and digital tools than individuals with bipolar disorder [76].

### 8.2. Data Protection and Privacy

A critical limitation for the large-scale implementation of mobile sensing technologies (continuous audio, location tracking, biometric data) arises from concerns about privacy and personal data security, which include sensitive conversations and individual movements.

Mobility data is particularly invasive, and analysis of 20,000 apps on Google Play reveals that 45% of users use unencrypted communications, with 23% of personal data transmitted over insecure channels. In this regard, promising solutions such as federated learning, on-device computing, and blockchain could be considered as priority directions for balancing clinical innovation and privacy protection in psychosis contexts [77].

Despite their promise, privacy-preserving approaches such as federated learning and on-device processing still face several challenges before clinical implementation. From a technical perspective, issues include model robustness, data heterogeneity, and computational constraints on edge devices. Ethically, questions remain regarding informed consent, data ownership, and transparency of algorithmic decision-making, particularly in vulnerable populations such as individuals with complex psychosis.

From a regulatory standpoint, harmonization with data protection frameworks and the establishment of standards for clinical validation are required. Clinical validation studies are needed to demonstrate that privacy-preserving systems maintain diagnostic accuracy and therapeutic effectiveness comparable to centralized models. Addressing these challenges will be essential to ensure safe, scalable, and ethically sound implementation.

## 9. A Vision for the Future: Toward a Fully Integrated Digital Rehabilitation Ecosystem

The future of mental health outlines a digital rehabilitation ecosystem that is fully integrated with different working methods: rehabilitation is an integral part of a personalized, multimodal, real-time process.

At the heart of this vision is the coordination between biosensors, wearables, and AI-driven feedback loops, capable of transforming passive information into proactive and adaptive actions. These technologies should not operate in isolation but should be permanently incorporated into the services offered by local authorities and into stepped-care models. The stepped-care model is a dynamic approach that organizes treatments according to the severity and complexity of the clinical case, optimizing resources and maximizing effectiveness [78].

In the coming years, research should focus on validating these digital tools and seeking to provide ethical infrastructures that guarantee digital equity, so that digitization becomes a tool for inclusion and not a further tool for disparity.

Immersive technologies such as virtual and augmented reality provide protected environments for training social and functional skills, while AI-assisted cognitive remediation delivers personalized, adaptive, and gamified neurocognitive interventions. Concurrently, m-Health applications and wearable sensors enable real-time monitoring of physiological and symptomatic data in everyday contexts, complemented by avatar-based therapies supporting social reintegration and hallucination management.

**Figure 4:**
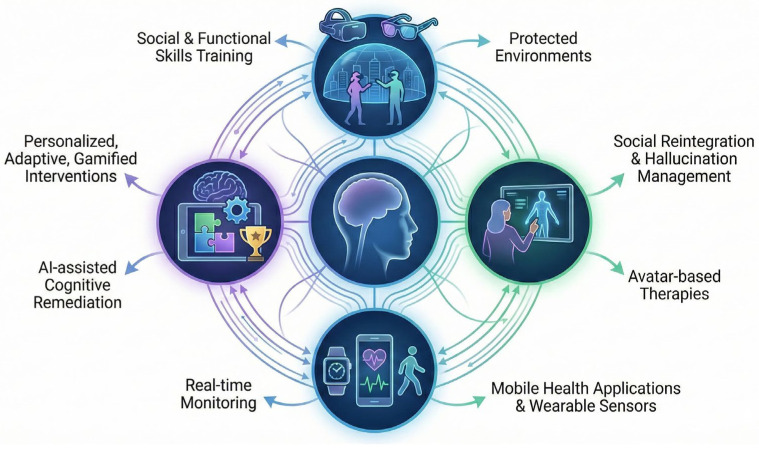
An integrated digital rehabilitation ecosystem for complex psychosis.

This integrated vision of future psychiatric rehabilitation is illustrated in Figure 4. This schematic illustration is intended to summarize potential system-level integration and does not reflect validated clinical models or quantitative data.

## 10. Limitations

Several limitations should be acknowledged. Although the literature search was structured, this manuscript was conceived as a narrative rather than a systematic review. Consequently, study identification and selection were guided by relevance and conceptual contribution rather than by formal systematic review procedures, no standardized quality appraisal or risk-of-bias tool was applied, and the possibility of selection bias cannot be ruled out.

The use of the term “complex psychosis” reflects a rehabilitation- and service-oriented perspective rather than a diagnostically or pathophysiologically homogeneous construct. This choice allowed us to address real-world psychiatric rehabilitation settings, but it also introduced substantial clinical heterogeneity, requiring caution in the interpretation of findings across diagnostic categories. Table 2 provides a conceptual overview of the clinical and functional features that justify its use in the present review.

The current evidence base is largely dominated by studies involving schizophrenia-spectrum populations; therefore, the extension of these findings to bipolar disorder with psychotic features, psychotic depression, or other psychotic conditions should be regarded as provisional.

The available literature remains methodologically uneven, with marked heterogeneity in study designs, sample sizes, intervention formats, outcome measures, and follow-up periods, and with many findings still derived from pilot or feasibility studies rather than robust confirmatory trials.

Finally, the broad scope of this review, while consistent with its integrative aim, necessarily limited the depth of critical appraisal within individual technological domains. All these limitations underscore the need for more rigorous, disorder-sensitive, and longitudinal research before firm conclusions can be drawn regarding the role of digital rehabilitation in complex psychosis.

## 11. Conclusions

The integration of advanced technologies such as VR, AI-guided CR, and m-Health monitoring is shaping a new paradigm in psychiatric rehabilitation. This digital ecosystem not only enables personalized, real-time interventions for symptoms that are often resistant to medication but also promotes a more efficient and accessible stepped-care model. While the broad scope of this review may limit the depth of analysis for individual technologies, it reflects the need for an integrated framework capable of capturing the multidimensional nature of digital rehabilitation in complex psychosis.

In the present review, findings should be interpreted with caution across diagnostic categories, as the transferability of evidence may vary according to the underlying disorder. The broader scope of this review was nonetheless maintained to reflect the complexity of real-world psychiatric rehabilitation settings, where digital interventions are often considered across diagnostically heterogeneous but functionally severe populations.

Overall, digital and immersive technologies represent promising adjuncts to psychiatric rehabilitation in complex psychosis, but it is pivotal to stress that across domains, the maturity of the evidence base is uneven: VR and computerized cognitive remediation are supported by a relatively more developed literature, whereas AR, digital phenotyping, and some AI-assisted approaches remain more dependent on preliminary or exploratory studies.

Importantly, many digital interventions show more consistent effects on process-related outcomes, such as engagement, adherence, usability, or continuity of monitoring, than on harder clinical or functional outcomes, such as relapse prevention, hospitalization, sustained autonomy, or real-world psychosocial functioning.

A major future challenge will be to determine which of these innovations can be translated into standardized clinical practice through rigorous validation, equitable implementation, and careful attention to socioeconomic barriers and continuity of care between clinical and everyday settings.

## Figures and Tables

**Table 1:** Digital rehabilitation techniques used in patients with complex psychosis.

Technology	Intervention Area	Key Function/Objectives
Virtual Reality and Augmented Reality	Social cognition and functioning, skill training and environmental support	Symptom management and Practical Training
Remote psychosocial interventions and Tele-cognitive remediation programs	Social cognition and remote cognitive programs	Continuity of care and neurocognition
Mobile Health	Psychoeducation and self-management	Apps for symptoms monitoring and prevention
Digital Phenotyping	Sensor-based monitoring	Analyzing passive sensing to detect crises
Artificial Intelligence and Gamification	Advanced cognitive remediation	Personalized training

**Table 2:** Clinical and rehabilitation features underlying the use of the term “complex psychosis” in this review.

Domain	Description	Examples	Relevance for Digital Rehabilitation
Diagnostic scope	The term complex psychosis is used as a pragmatic, service-oriented construct rather than a formal diagnostic category	Schizophrenia-spectrum disorders, bipolar disorder with psychotic features, psychotic depression, schizoaffective presentations, persistent delusional conditions	Supports a rehabilitation-focused rather than diagnosis-restricted discussion of digital interventions
Functional severity	Patients typically present marked impairment in everyday functioning and autonomy	Difficulties in self-care, social functioning, work or study participation, independent living	Justifies interventions targeting daily functioning, skills training, and recovery support
Symptom persistence	Clinical complexity often includes persistent psychotic symptoms and partial response to standard treatment	Residual positive symptoms, negative symptoms, treatment resistance, recurrent instability	Supports the exploration of adjunctive and personalized rehabilitation tools
Cognitive impairment	Cognitive deficits are frequently present and clinically relevant	Attention, memory, executive dysfunction, social cognition deficits	Provides rationale for cognitive remediation, Tele-CR, gamified training, and VR-based approaches
Multimorbidity and complexity	Complexity often includes psychiatric, developmental, substance-related, or physical comorbidities	Substance misuse, ASD/ADHD traits, anxiety, metabolic and cardiovascular burden	Highlights the need for flexible, multimodal, and scalable rehabilitation pathways
High support needs	Patients often require structured, multidisciplinary, or long-term care pathways	Recurrent hospitalizations, supported housing, forensic pathways, early intervention transitions	Reflects real-world service settings in which digital tools may complement ongoing rehabilitation
Conceptual limitation	The construct is clinically useful but not diagnostically homogeneous	Evidence may not transfer equally across all psychotic disorders	Requires cautious interpretation and limits overgeneralization

Abbreviations: ADHD, attention-deficit/hyperactivity disorder; ASD, autism spectrum disorder; Tele-CR, tele-cognitive remediation; VR, virtual reality.

## Data Availability

No new data were created or analyzed in this study. All data are in the manuscript and in cited articles.

## Abbreviations

The following abbreviations are used in this manuscript:

CP	Complex Psychosis
ASD	Autism Spectrum Disorder
ADHD	Attention Deficit Hyperactivity Disorder
SAMHSA	Substance Abuse and Mental Health Services Administration
CBT	Cognitive Behavioral Therapy
VR	Virtual Reality
AR	Augmented Reality
CR	Cognitive Remediation
VRT	Virtual Reality Therapy
SST	Social Skill Training
Tele-CR	Tele-Cognitive Remediation
m-Health	Mobile-Health
AT	Avatar Therapy
EMA	Ecological Momentary Assessments
CCR	Computerized Cognitive Remediation
GDNF	Glial Derived Neurotrophic Factor
MCR	Manual Cognitive Remediation
AI	Artificial Intelligence

## References

[1] Kim K., Jeon H.J., Myung W., Suh S.W., Seong S.J., Hwang J.Y., Ryu J.I., Park S.C. (2022). Clinical Approaches to Late-Onset Psychosis. J. Pers. Med..

[2] National Institute for Health and Care Excellence (NICE) (2020). Rehabilitation for Adults with Complex Psychosis.

[3] Killaspy H., Craig T., Dark F., Harvey C., Medalia A. (2021). Editorial: Design and Implementation of Rehabilitation Interventions for People with Complex Psychosis. Front. Psychiatry.

[4] Killaspy H. (2014). The ongoing need for local services for people with complex mental health problems. Psychiatr. Bull..

[5] López-Carrilero R., Lo Monaco M., Frígola-Capell E., Ferrer-Quintero M., Díaz-Cutraro L., Verdaguer-Rodríguez M., García-Mieres H., Vila-Badia R., Punsoda-Puche P., Birulés I. (2024). Cognitive insight in first-episode psychosis: Exploring the complex relationship between executive functions and social cognition. Span. J. Psychiatry Ment. Health.

[6] Zhang X., Lewis S., Chen X., Berry N., Bucci S. (2023). Technology use and attitudes towards digital mental health in people with severe mental health problems: A survey study in China. Front. Psychiatry.

[7] Robson E., Greenwood K. (2022). Rates and Predictors of Disengagement and Strength of Engagement for People with a First Episode of Psychosis Using Early Intervention Services: A Systematic Review of Predictors and Meta-analysis of Disengagement Rates. Schizophr. Bull. Open.

[8] Agley J., Gassman R., Reho K., Roberts J., Heil S.K.R., Golzarri-Arroyo L., Eddens K. (2024). Organizational Network Analysis of SAMHSA’s Technology Transfer Center (TTC) Network. J. Behav. Health Serv. Res..

[9] Farhall J., Castle D., Constantine E., Foley F., Kyrios M., Rossell S., Arnold C., Leitan N., Villagonzalo K.A., Brophy L. (2023). Using a digital personal recovery resource in routine mental health practice: Feasibility, acceptability and outcomes. J. Ment. Health.

[10] Revell E.R., Neill J.C., Harte M., Khan Z., Drake R.J. (2015). A systematic review and meta-analysis of cognitive remediation in early schizophrenia. Schizophr. Res..

[11] Bowie C.R. (2019). Cognitive remediation for severe mental illness: State of the field and future directions. World Psychiatry.

[12] Prensky M. (2001). Digital natives, digital immigrants part 1. Horiz..

[13] Ben-Zeev D., Buck B., Kopelovich S., Meller S. (2019). A technology-assisted life of recovery from psychosis. NPJ Schizophr..

[14] Camacho E., Torous J. (2021). Interest and readiness for digital mental health in coordinate specialty care for early course psychosis: A survey study of 42 programs in 30 states. Early Interv. Psychiatry.

[15] Singh S.P., Mohan M., Giacco D. (2021). Psychosocial interventions for people with a first episode psychosis: Between tradition and innovation. Curr. Opin. Psychiatry.

[16] Bird M., O’Neill E., Riches S. (2024). Digitally Enhanced Psychological Assessment and Treatment of Paranoia: A Systematic Review. Clin. Psychol. Psychother..

[17] Vita A., Barlati S., Ceraso A., Nibbio G., Ariu C., Deste G., Wykes T. (2021). Effectiveness, Core Elements, and Moderators of Response of Cognitive Remediation for Schizophrenia: A Systematic Review and Meta-analysis of Randomized Clinical Trials. JAMA Psychiatry.

[18] Wykes T., Spaulding W.D. (2011). Thinking about the future cognitive remediation therapy—What works and could we do better?. Schizophr. Bull..

[19] Harvey P.D., McGurk S.R., Mahncke H., Wykes T. (2018). Controversies in Computerized Cognitive Training. Biol. Psychiatry Cogn. Neurosci. Neuroimaging.

[20] Kambeitz-Ilankovic L., Betz L.T., Dominke C., Haas S.S., Subramaniam K., Fisher M., Vinogradov S., Koutsouleris N., Kambeitz J. (2019). Multi-outcome meta-analysis (MOMA) of cognitive remediation in schizophrenia: Revisiting the relevance of human coaching and elucidating interplay between multiple outcomes. Neurosci. Biobehav. Rev..

[21] Cella M., Price T., Corboy H., Onwumere J., Shergill S., Preti A. (2020). Cognitive remediation for inpatients with psychosis: A systematic review and meta-analysis. Psychol. Med..

[22] Medalia A., Beck A.T., Grant P.M. (2019). Cognitive therapies for psychosis: Advances and challenges. Schizophr. Res..

[23] Corbera S., Wexler B.E., Poltorak A., Thime W.R., Kurtz M.M. (2017). Cognitive remediation for adults with schizophrenia: Does age matter?. Psychiatry Res..

[24] Cipresso P., Giglioli I.A.C., Raya M.A., Riva G. (2018). The Past, Present, and Future of Virtual and Augmented Reality Research: A Network and Cluster Analysis of the Literature. Front. Psychol..

[25] Srivastava K., Das R.C., Chaudhury S. (2014). Virtual reality applications in mental health: Challenges and perspectives. Ind. Psychiatry J..

[26] Machado S., Paes F., Ferreira-Garcia R., Gonçalves L.L., Carta M.G., Appolinario J.C., Nardi A.E. (2025). Virtual Reality: Challenges and Perspectives in Mental Health. Alpha Psychiatry.

[27] Cushnan J., McCafferty P., Best P. (2024). Clinicians’ perspectives of immersive tools in clinical mental health settings: A systematic scoping review. BMC Health Serv. Res..

[28] Park K.M., Ku J., Choi S.H., Jang H.J., Park J.Y., Kim S.I., Kim J.J. (2011). A virtual reality application in role-plays of social skills training for schizophrenia: A randomized, controlled trial. Psychiatry Res..

[29] Dellazizzo L., Percie du Sert O., Phraxayavong K., Potvin S., O’Connor K., Dumais A. (2018). Exploration of the dialogue components in Avatar Therapy for schizophrenia patients with refractory auditory hallucinations: A content analysis. Clin. Psychol. Psychother..

[30] Park M.J., Kim D.J., Lee U., Na E.J., Jeon H.J. (2019). A Literature Overview of Virtual Reality (VR) in Treatment of Psychiatric Disorders: Recent Advances and Limitations. Front. Psychiatry.

[31] Moritz S., Menon M., Balzan R., Woodward T.S. (2023). Metacognitive training for psychosis (MCT): Past, present, and future. Eur. Arch. Psychiatry Clin. Neurosci..

[32] Pot-Kolder R., Veling W., Geraets C., Lokkerbol J., Smit F., Jongeneel A., Ising H., van der Gaag M. (2020). Cost-effectiveness of virtual reality cognitive behavioral therapy for psychosis: Health-economic evaluation within a randomized controlled trial. J. Med. Internet Res..

[33] Percie du Sert O., Potvin S., Lipp O., Dellazizzo L., Laurelli M., Breton R., Lalonde P., Phraxayavong K., O’Connor K., Pelletier J.F. (2018). Virtual reality therapy for refractory auditory verbal hallucinations in schizophrenia: A pilot clinical trial. Schizophr. Res..

[34] Freeman D. (2024). Developing psychological treatments for psychosis. Br. J. Psychiatry.

[35] Rus-Calafell M., Schneider S. (2020). Are we there yet?!—A literature review of recent digital technology advances for the treatment of early psychosis. Mhealth.

[36] Jahn F.S., Skovbye M., Obenhausen K., Jespersen A.E., Miskowiak K.W. (2021). Cognitive training with fully immersive virtual reality in patients with neurological and psychiatric disorders: A systematic review of randomized controlled trials. Psychiatry Res..

[37] Murali S., Paul K.D., McGwin G., Ponce B.A. (2021). Updates to the Current Landscape of Augmented Reality in Medicine. Cureus.

[38] Bell I., Pot-Kolder R.M.C.A., Wood S.J., Nelson B., Acevedo N., Stainton A., Nicol K., Kean J., Bryce S., Bartholomeusz C.F. (2022). Digital technology for addressing cognitive impairment in recent-onset psychosis: A perspective. Schizophr. Res. Cogn..

[39] Lan L., Sikov J., Lejeune J., Ji C., Brown H., Bullock K., Spencer A.E. (2023). A Systematic Review of using Virtual and Augmented Reality for the Diagnosis and Treatment of Psychotic Disorders. Curr. Treat. Options Psychiatry.

[40] Amadeo M.B., Esposito D., Escelsior A., Campus C., Inuggi A., Pereira Da Silva B., Serafini G., Amore M., Gori M. (2022). Time in schizophrenia: A link between psychopathology, psychophysics and technology. Transl. Psychiatry.

[41] Turner D.T., McGlanaghy E., Cuijpers P., van der Gaag M., Karyotaki E., MacBeth A. (2018). A Meta-Analysis of Social Skills Training and Related Interventions for Psychosis. Schizophr. Bull..

[42] Hache-Labelle C., Abdel-Baki A., Lepage M., Laurin A.S., Guillou A., Francoeur A., Bergeron S., Lecomte T. (2021). Romantic relationship group intervention for men with early psychosis: A feasibility, acceptability and potential impact pilot study. Early Interv. Psychiatry.

[43] Lecomte T., Abdel-Baki A., Francoeur A., Cloutier B., Leboeuf A., Abadie P., Villeneuve M., Guay S. (2021). Group therapy via videoconferencing for individuals with early psychosis: A pilot study. Early Interv. Psychiatry.

[44] Santesteban-Echarri O., Piskulic D., Nyman R.K., Addington J. (2020). Telehealth interventions for schizophrenia-spectrum disorders and clinical high-risk for psychosis individuals: A scoping review. J. Telemed. Telecare.

[45] Lynch D.A., Medalia A., Saperstein A. (2020). The Design, Implementation, and Acceptability of a Telehealth Comprehensive Recovery Service for People with Complex Psychosis Living in NYC During the COVID-19 Crisis. Front. Psychiatry.

[46] Thibaudeau E., Peyroux E., Franck N., Carling H., Lepage M. (2024). Navigating Social Cognitive Impairments in Schizophrenia Spectrum Disorders: Protocol for a Pilot Pre-Post Quasi-Experimental Study for Remote Avatar-Assisted Cognitive Remediation Therapy. JMIR Res. Protoc..

[47] McEnery C., Lim M.H., Knowles A., Rice S., Gleeson J., Howell S., Russon P., Miles C., D’Alfonso S., Alvarez-Jimenez M. (2021). Social anxiety in young people with first-episode psychosis: Pilot study of the EMBRACE moderated online social intervention. Early Interv. Psychiatry.

[48] Smith K., Ostinelli E., Macdonald O., Cipriani A. (2020). COVID-19 and Telepsychiatry: Development of Evidence-Based Guidance for Clinicians. JMIR Ment. Health.

[49] Daele T.V., Karekla M., Kassianos A.P., Compare A., Haddouk L., Salgado J., Ebert D.D., Trebbi G., Bernaerts S., Van Assche E. (2020). Recommendations for policy and practice of telepsychotherapy and e-mental health in Europe and beyond. J. Psychother. Integr..

[50] Davies K., Grattan S., Gott C., Ellis R., Lappin J.M. (2023). The tertiary service for psychosis: Holistic recommendations for people with complex psychosis. Australas Psychiatry.

[51] Cella M., Parri L., Wang K., Quinn R., Oyeleye O., Jin H., Wykes T. (2024). Evaluating remote delivery of cognitive remediation in people with psychosis. Schizophr. Res..

[52] Cella M., Wykes T. (2019). The nuts and bolts of Cognitive Remediation: Exploring how different training components relate to cognitive and functional gains. Schizophr. Res..

[53] Reeder C., Huddy V., Cella M., Taylor R., Greenwood K., Landau S., Wykes T. (2017). A new generation computerised metacognitive cognitive remediation programme for schizophrenia (CIRCuiTS): A randomised controlled trial. Psychol. Med..

[54] Chirokoff V., Tessier A., Serre F., Dupuy M., Auriacombe M., Chanraud S., Berthoz S., Fatseas M., Misdrahi D. (2025). Relevance of ecological momentary assessment for medication adherence in clinical settings: A precision psychiatry approach. Br. J. Clin. Psychol..

[55] Skoge M., Aminoff S.R., Barrett E.A., Bryhni G.E., Kling K., Kværner K.J., Melle I., Mork E., Simonsen C., Støme L.N. (2025). A Mobile App Designed to Promote Shared Decision-Making in the Treatment of Psychotic Disorders: Feasibility and Acceptability Study. JMIR Hum. Factors.

[56] Fulford D., Mote J., Gonzalez R., Abplanalp S., Zhang Y., Luckenbaugh J., Onnela J.P., Busso C., Gard D.E. (2021). Smartphone sensing of social interactions in people with and without schizophrenia. J. Psychiatr. Res..

[57] Gumley A.I., Bradstreet S., Ainsworth J., Allan S., Alvarez-Jimenez M., Aucott L., Birchwood M., Briggs A., Bucci S., Cotton S.M. (2022). The EMPOWER blended digital intervention for relapse prevention in schizophrenia: A feasibility cluster randomised controlled trial in Scotland and Australia. Lancet Psychiatry.

[58] Hassan L., Milton A., Sawyer C., Casson A.J., Torous J., Davies A., Ruiz-Yu B., Firth J. (2025). Utility of Consumer-Grade Wearable Devices for Inferring Physical and Mental Health Outcomes in Severe Mental Illness: Systematic Review. JMIR Ment. Health.

[59] Zhang M., Chen X., Yao Y., Wang W., Zhong Y., Shi S., Zhang K. (2025). Smartphone video games effectively improve cognitive function in middle-aged and elderly patients with chronic schizophrenia: A randomized clinical trial. Transl. Psychiatry.

[60] Bladon S., Eisner E., Bucci S., Oluwatayo A., Martin G.P., Sperrin M., Ainsworth J., Faulkner S. (2025). A systematic review of passive data for remote monitoring in psychosis and schizophrenia. NPJ Digit. Med..

[61] Raugh I.M., James S.H., Gonzalez C.M., Chapman H.C., Cohen A.S., Kirkpatrick B., Strauss G.P. (2021). Digital phenotyping adherence, feasibility, and tolerability in outpatients with schizophrenia. J. Psychiatr. Res..

[62] Cohen A., Joshi D., Bondre A., Chand P.K., Chaturvedi N., Choudhary S., Dutt S., Khan A., Langholm C., Kumar M. (2024). Digital phenotyping correlates of mobile cognitive measures in schizophrenia: A multisite global mental health feasibility trial. PLoS Digit. Health.

[63] Barnett I., Torous J., Staples P., Sandoval L., Keshavan M., Onnela J.P. (2018). Relapse prediction in schizophrenia through digital phenotyping: A pilot study. Neuropsychopharmacology.

[64] Adler D.A., Ben-Zeev D., Tseng V.W., Kane J.M., Brian R., Campbell A.T., Hauser M., Scherer E.A., Choudhury T. (2020). Predicting Early Warning Signs of Psychotic Relapse from Passive Sensing Data: An Approach Using Encoder-Decoder Neural Networks. JMIR mHealth uHealth.

[65] Bufano P., Laurino M., Said S., Tognetti A., Menicucci D. (2023). Digital Phenotyping for Monitoring Mental Disorders: Systematic Review. J. Med. Internet Res..

[66] Bell I.H., Eisner E., Allan S., Cartner S., Torous J., Bucci S., Thomas N. (2024). Methodological Characteristics and Feasibility of Ecological Momentary Assessment Studies in Psychosis: A Systematic Review and Meta-Analysis. Schizophr. Bull..

[67] Depp C.A., Kamarsu S., Filip T.F., Parrish E.M., Harvey P.D., Granholm E.L., Chalker S., Moore R.C., Pinkham A. (2022). Ecological momentary facial emotion recognition in psychotic disorders. Psychol. Med..

[68] Zhang P., Chen L., Qin Q., Liu C., Zhu H., Hu W., He X., Tang K., Yan Q., Shen H. (2025). Enhanced computerized cognitive remediation therapy improved cognitive function, negative symptoms, and GDNF in male long-term inpatients with schizophrenia. Front. Psychiatry.

[69] Hu J.J., Sun X.R., Ni S.M., Kong Y. (2024). Computerized cognitive remediation therapy on cognitive impairment and social function in patients with chronic schizophrenia. World J. Psychiatry.

[70] Yee J., Matheson H., Bogie B.J.M., Du Perron É., Thérond A., Charest M., van Driel C., Goyette M., Lei Y.T., Noël C. (2025). Cognitive Remediation for Psychosis in Virtual Reality (ThinkTactic VR): Qualitative, Iterative, and User-Centered Codevelopment Study. JMIR Ment Health.

[71] Perra A., Riccardo C.L., De Lorenzo V., De Marco E., Di Natale L., Kurotschka P.K., Preti A., Carta M.G. (2023). Fully Immersive Virtual Reality-Based Cognitive Remediation for Adults with Psychosocial Disabilities: A Systematic Scoping Review of Methods Intervention Gaps and Meta-Analysis of Published Effectiveness Studies. Int. J. Environ. Res. Public Health.

[72] Wang J., Zhang J., Xu P., Qian T., Tan S., Liang P. (2024). Is game-based therapy effective for treating cognitive deficits in adults with schizophrenia? Evidence from a randomized controlled trial. Transl. Psychiatry.

[73] Plechatá A., Hejtmánek L., Bednářová M., Fajnerová I. (2021). Cognitive Remediation in Virtual Environments for Patients with Schizophrenia and Major Depressive Disorder: A Feasibility Study. Int. J. Environ. Res. Public Health.

[74] Zhu X., Fan H., Zou Y., Tan Y., Yang F., Wang Z., Zhao Y., Fan F., Reeder C., Zhou D. (2022). Computerized or manual? Long term effects of cognitive remediation on schizophrenia. Schizophr. Res..

[75] Fond G.B., Yon D.K., Tran B., Mallet J., Urbach M., Leignier S., Rey R., Misdrahi D., Llorca P.M., Schürhoff F. (2023). Poverty and inequality in real-world schizophrenia: A national study. Front. Public Health.

[76] Paquin V., Ackerman R.A., Depp C.A., Moore R.C., Harvey P.D., Pinkham A.E. (2024). Media Use and Its Associations with Paranoia in Schizophrenia and Bipolar Disorder: Ecological Momentary Assessment. JMIR Ment. Health.

[77] Dang T., Spathis D., Ghosh A., Mascolo C. (2023). Human-centred artificial intelligence for mobile health sensing: Challenges and opportunities. R. Soc. Open Sci..

[78] Cosgrove J.A., Crocker L.D., Breitborde N.J.K., Rosenblatt A., George P., Marshall T., Lichvar E., Mullins J., Schiffman J. (2026). Stepped Care Interventions for Psychosis Risk: Findings from Clinical High Risk for Psychosis Grantee Programs. Psychiatr. Serv..

